# Molecular Characterization of the Human Stomach Microbiota in Gastric Cancer Patients

**DOI:** 10.3389/fcimb.2017.00302

**Published:** 2017-07-06

**Authors:** Guoqin Yu, Javier Torres, Nan Hu, Rafael Medrano-Guzman, Roberto Herrera-Goepfert, Michael S. Humphrys, Lemin Wang, Chaoyu Wang, Ti Ding, Jacques Ravel, Philip R. Taylor, Christian C. Abnet, Alisa M. Goldstein

**Affiliations:** ^1^Integrative Tumor Epidemiology Branch, Division of Cancer Epidemiology and Genetics, National Cancer Institute, National Institutes of HealthBethesda, MD, United States; ^2^Unidad de Investigacion en Enfermedades Infecciosas, Unidad Medica de Alta Especialidad Pediatria, Centro Medico Nacional SXXI, Instituto Mexicano del Seguro SocialMexico City, Mexico; ^3^Metabolic Epidemiology Branch, Division of Cancer Epidemiology and Genetics, National Cancer Institute, National Institutes of HealthBethesda, MD, United States; ^4^Unidad Medica de Alta Especialidad Oncología, Centro Medico Nacional SXXI, Instituto Mexicano del Seguro SocialMexico City, Mexico; ^5^Instituto Nacional de Cancerología, Secretaria de SalúdMexico City, Mexico; ^6^Institute for Genome Sciences, University of Maryland School of MedicineBaltimore, MD, United States; ^7^Shanxi Cancer HospitalTaiyuan, China; ^8^Clinical Genetics Branch, Division of Cancer Epidemiology and Genetics, National Cancer Institute, National Institutes of HealthBethesda, MD, United States

**Keywords:** *Helicobacter pylori*, 16S rRNA, KEGG modules, microbiome, gastric cancer

## Abstract

*Helicobacter pylori* (*Hp*) is the primary cause of gastric cancer but we know little of its relative abundance and other microbes in the stomach, especially at the time of gastric cancer diagnosis. Here we characterized the taxonomic and derived functional profiles of gastric microbiota in two different sets of gastric cancer patients, and compared them with microbial profiles in other body sites. Paired non-malignant and tumor tissues were sampled from 160 gastric cancer patients with 80 from China and 80 from Mexico. The 16S rRNA gene V3–V4 region was sequenced using MiSeq platform for taxonomic profiles. PICRUSt was used to predict functional profiles. Human Microbiome Project was used for comparison. We showed that *Hp* is the most abundant member of gastric microbiota in both Chinese and Mexican samples (51 and 24%, respectively), followed by oral-associated bacteria. Taxonomic (phylum-level) profiles of stomach microbiota resembled oral microbiota, especially when the *Helicobacter* reads were removed. The functional profiles of stomach microbiota, however, were distinct from those found in other body sites and had higher inter-subject dissimilarity. Gastric microbiota composition did not differ by *Hp* colonization status or stomach anatomic sites, but did differ between paired non-malignant and tumor tissues in either Chinese or Mexican samples. Our study showed that *Hp* is the dominant member of the non-malignant gastric tissue microbiota in many gastric cancer patients. Our results provide insights on the gastric microbiota composition and function in gastric cancer patients, which may have important clinical implications.

## Background

Gastric cancer (GC) is the fifth most common cancer in the world and the third leading cause of cancer death (Ferlay et al., 2013). GC incidence varies widely with high rates in Asia, Eastern Europe, and Central and South America, and low rates in North America and Africa (Carneiro, 2014). GC may arise in cardia or in non-cardia (the fundus, body, or pylorus section). Chronic colonization of *Helicobacter pylori (Hp)* is known to increase the risk of non-cardia cancer (Cavaleiro-Pinto et al., 2011). The association between *Hp* colonization and gastric cardia cancer varies by populations. The studies in Western countries tend to show a neutral or even negative association while in Eastern populations namely China, Japan, and Korea, there is strong evidence of a higher risk of cardia cancer among subjects with *Hp* colonization (Cavaleiro-Pinto et al., 2011).

Chronic inflammation of the stomach may progress through a series of steps including atrophic gastritis, intestinal metaplasia, dysplasia, and gastric adenocarcinoma (Correa, 2013). Atrophic gastritis, the loss of specialized glandular tissue with impaired acid secretion and differentiation of gastric progenitor cells, results in hypochlorhydria in the stomach. It is generally believed that *Hp* prefers a healthy gastric mucosa and that as the steps to GC progress, *Hp* is also gradually fading, until it disappears. Therefore, the stomachs of patients with GC should facilitate the colonization of the gastric mucosa by bacteria other than *Hp* (Sheh and Fox, 2013). Studies of gastric microbiota are sparse. Previous studies were often small, not in GC patients or used biopsy samples collected during endoscopy, which may have led to contamination from the oral cavity (see Supplementary Table 1 for summary of previous studies). Therefore, gastric microbiota in GC patients remains largely unknown.

Chronic colonization of *Hp* is the major risk factor for GC in both Chinese and Mexican populations (Kamangar et al., 2007; Ayala et al., 2011). However, GC occurs mainly at the cardia of the stomach in Shanxi, China, but in the non-cardia of the stomach in Mexico. In this study, we profiled the taxonomic and functional profiles in non-malignant gastric tissue from two collections of GC patients separately, one from China (cardia cancer cases) and the second from Mexico (non-cardia cancer cases). We compared gastric non-malignant tissue with paired tumor tissues and with other body sites including oral, nasal cavity, stool, vagina, and skin using data from the Human Microbiome Project (HMP) (Human Microbiome Project Consortium, 2012). We also evaluated differences in the gastric microbiota by *Hp* colonization status, anatomical sites within the stomach for the non-cardia cancer samples, and tissue type (non-malignant and tumor) separately for the two sample populations.

## Materials and methods

### Study subjects and sample collection

The Chinese gastric tissue samples were from 80 gastric cardia cancer patients recruited at the Shanxi Cancer Hospital in Taiyuan, Shanxi Province, China, between 1998 and 2001. This study was approved by the Institutional Review Boards of the Shanxi Cancer Hospital and the National Cancer Institute (NCI). All subjects provided written informed consent prior to participation. Cases were histologically confirmed as adenocarcinomas by pathologists at both the Shanxi Cancer Hospital and the NCI. Clinical data was collected by review of medical records. Patients who were <18 years old, with cancer other than GC or with previous treatment for GC were excluded. Tumor tissues and matched non-malignant tissues distant to the tumor were obtained from surgical resections, snap frozen in liquid nitrogen, and stored at −130°C until used. H&E slides were used to determine the percentage of tumor cells in the tissues. Total DNA was extracted using the Allprep RNA/DNA/Protein mini kit (QIAGEN) following the protocol provided by the manufacturer.

The Mexican gastric tissue samples were from 80 gastric non-cardia cancer patients recruited at the Oncology Hospital, Centro Médico Nacional Siglo XXI, Instituto Mexicano del Seguro Social, and the Instituto Nacional de Cancerología, Secretaria de Salud in Mexico City, Mexico, between 2008 and 2013. The study was approved by the ethics committee of each hospital and written informed consent was obtained from all patients prior to enrollment in the study. Cases were histologically confirmed by the pathologist. The clinical and pathological data were recorded in questionnaires. Patients who were <18 years old, with any autoimmune disease, diabetes, or cancer other than GC, and with a previous treatment for GC were excluded from the study. Tumor tissue and matched non-malignant tissue distant to the tumor were obtained from surgical resection specimens, placed immediately in microfuge tubes and submerged in a container with liquid nitrogen, and stored at −70°C until tested. H&E slides were used to determine the percentage of tumor cells in the tissues. Total DNA was extracted by QIAamp DNA mini kit (QIAGEN) using the protocol provided by the manufacturer.

All the non-malignant tissue samples were verified with absence of tumor cells. Tumor tissue samples without tumor cells were excluded from all analyses. The percentage of tumor cells were 70–80% in the Chinese tumor samples and 30–50% in Mexican tumor samples. Examples of H& E slides are shown in Supplementary Figure 1.

### 16S rRNA gene sequence analysis

The V3–V4 region of the 16S rRNA gene was amplified and sequenced on the Illumina MiSeq platform using the 300 paired-end protocol at the Institute of Genome Sciences, University of Maryland School of Medicine as described previously (Fadrosh et al., 2014).

Sequence reads were processed to remove low quality, short, or chimera reads (Yu et al., 2015). We removed low quality reads (reads with average quality <20 over 30 bp window based on Phred algorithm; paired reads which have at least one read with length <75% of its original length) and chimera reads (by UCHIME). The remaining reads with at least 97% sequence identity were clustered into species-level Operational Taxonomy Units (OTUs) in the software package Quantitative Insights into Microbial Ecology (QIIME 1.8.0) (Caporaso et al., 2010) by using command pick_open_reference_otus.py with usearch61 clustering algorithm and other default settings. The OTUs were assigned to taxa (e.g., genus, family, phylum) using the Greengenes database as reference (version 13_8; DeSantis et al., 2006). OTUs with only one read were excluded from analysis. Samples with <1,000 reads were excluded from analysis. The sequence data were submitted to BioProject database (accession number of 310127) at the National Center for Biotechnology Information website.

Alpha diversity was estimated as number of OTUs, Shannon's Index (Shannon, 1997), and Phylogenetic diversity (PD_whole_tree) (Faith and Baker, 2006) by averaging over 20 rarefied tables of 1,000 reads/sample. Alpha diversity was used to measure the species diversity of each sample. The number of OTUs, also known as richness, is a measure of diversity that does not consider the frequency of OTUs. Shannon's index is estimated by both the number and frequency of the OTUs. PD_whole_tree further takes account of the phylogenetic relationship of OTUs. The phylogenetic tree of OTUs used for PD_whole-tree estimates was prepared in QIIME based on neighbor-joining method. The alpha diversity increased with number of sequence reads sampled (Supplementary Figure 2). The alpha diversity showed differences by sample groups with 1,000 reads/sample; the order of sample groups based on alpha diversity did not change by number of sequence reads.

Beta diversity was measured as unweighted (presence/absence of taxa) and weighted (using taxa relative abundance information) UniFrac distance (Lozupone et al., 2011). Beta diversity measures dissimilarities of two samples in microbial profiles. We calculated both alpha and beta diversity based on rarefied tables of 1,000 reads/sample.

The relative abundance of taxa at different levels (phylum, class, order, family, and genus) was calculated based on the unrarefied table. The taxa relative abundance was estimated as the proportion of OTUs assigned to a taxon.

The Human Microbiome Project (HMP) 16S rRNA V3–V5 data were downloaded for comparison (http://hmpdacc.org/HMQCP/; Human Microbiome Project Consortium, 2012). The HMP sequence reads were processed in the same manner as described above. A total of 2,579 samples from 5 body sites (including oral, nasal cavity, stool, vagina, and skin) of 242 healthy US adults in HMP phase 1 were used for comparison (Aagaard et al., 2013). The study by Lozupone et al. (2013) showed that the difference in population or technologies used should not affect the comparison by body sites. In addition, we limited the comparison of HMP and stomach microbiota data to the highest and least variable taxonomic (phylum)/functional (module) level so that the population/technology differences between these two studies would have limited effect on the comparisons.

### Metagenomic prediction

We used Phylogenetic Investigation of Communities by Reconstruction of Unobserved States (PICRUSt)1.0.0 (Langille et al., 2013) to predict virtual metagenomes for each sample from the 16S rRNA gene sequence data and used the KEGG database as a reference (Kanehisa et al., 2014) to determine the relative abundance of metabolic pathways and modules within the virtual metagenomes. PICRUSt requires the use of Greengenes reference, version 13_5 to cluster reads into OTUs (DeSantis et al., 2006). Therefore, we re-clustered all the sequence data (including HMP) in QIIME with the command parallel_pick_otus_usearch61_ref.py and the Greengenes reference version 13_5.

### Statistical analysis

The Wilcoxon rank-sum test was used to examine gastric microbiota alpha diversity and taxa relative abundance differences between antrum and corpus in Mexican samples or between *Hp*+ and *Hp*− samples. Wilcoxon signed-rank test was used for the differences between non-malignant and matched tumor samples in each population. When examining taxa relative abundance, Bonferroni correction was used to adjust for tests of multiple taxa. Permutational Multivariate Analysis of Variance (PERMANOVA, adonis) was used to compare sample groups by unweighted/weighted UniFrac distance matrix. *P* < 0.05 were considered significant after adjustment for multiple tests.

In order to compare gastric microbiota with the oral, nasal, stool, skin, and vagina microbiota from the HMP study, we calculated Euclidean/Bray-Curtis distances and generated a matrix from the phylum-level/KEGG module profiles, performed principal coordinate analysis (PCoA) on the Euclidean/Bray-Curtis distance matrix, and then plotted the figure based on the first three principal coordinates to visualize the similarities and differences among different body sites. All statistical analyses were performed in R. Bray-Curtis and Euclidean distance showed similar results, therefore only Bray-Curtis distance is shown.

### Quality control

To address the concerns about possible contamination, we included 2 blank samples as negative controls. We also included 1 vaginal and 1 stool sample as positive controls to evaluate the performance of DNA amplification and sequencing. The two positive controls generated 2,703 and 58,201 reads, respectively, suggesting good performance of DNA amplification and sequencing. The blanks had extremely low number of reads (41 and 43 reads/sample, respectively). Furthermore, the OTUs found in both blanks were extremely rare in the gastric samples with accumulated relative abundance range of 0–0.006. Therefore, our results were unlikely to have been affected by contamination.

The conventional DNA extraction method for microbiome studies often includes an extra cell lysis step (bead-beating) to break the hard-to-break cell membranes of some species. To examine whether some taxa were missed due to the lack of a bead-beating step in our DNA extraction protocols, we evaluated 2 Chinese tissue samples using two different DNA extraction methods: a DNA extraction method with a bead-beating step and commonly used for microbiome study (Flores et al., 2012) and the method used for our Chinese samples in the current study. We found 14 genus-level taxa discovered by the extraction method with the bead-beating step that were not discovered by our DNA extraction method (Supplementary Table 2). However, these taxa were extremely rare with total cumulated relative abundance of 0.007 and 0.038 for two samples, respectively. Therefore, the DNA extraction method should not have adversely affected our findings, although we cannot exclude missing some rare taxa.

## Results

### Characteristics of the study subjects

After excluding samples with <1,000 reads per sample, 77 non-malignant gastric tissue samples from China and 80 from Mexico were included for analysis and the median (interquartile range) was 10,460 (5,454–19,980) reads per sample. The raw and qualified number of reads for each sample group are shown in Supplementary Table 3. The average age of these Chinese cases was 60.8 years old, 83% were male, and all were diagnosed with gastric cardia adenocarcinoma. The average age of Mexican cases was 64.5 years old, 54% were male, and all tumors were located in the non-cardia regions of the stomach (21 antrum, 24 corpus, 35 unspecified). In addition, 80 tumor samples from China and 54 from Mexico were also included for comparison [median (interquartile range): 9,406 (4,228–15,330) reads/sample] after excluding samples with <1,000 reads per sample or no tumor cells.

### Taxonomic and functional profiles of non-malignant gastric microbiota

**Figure 1:**
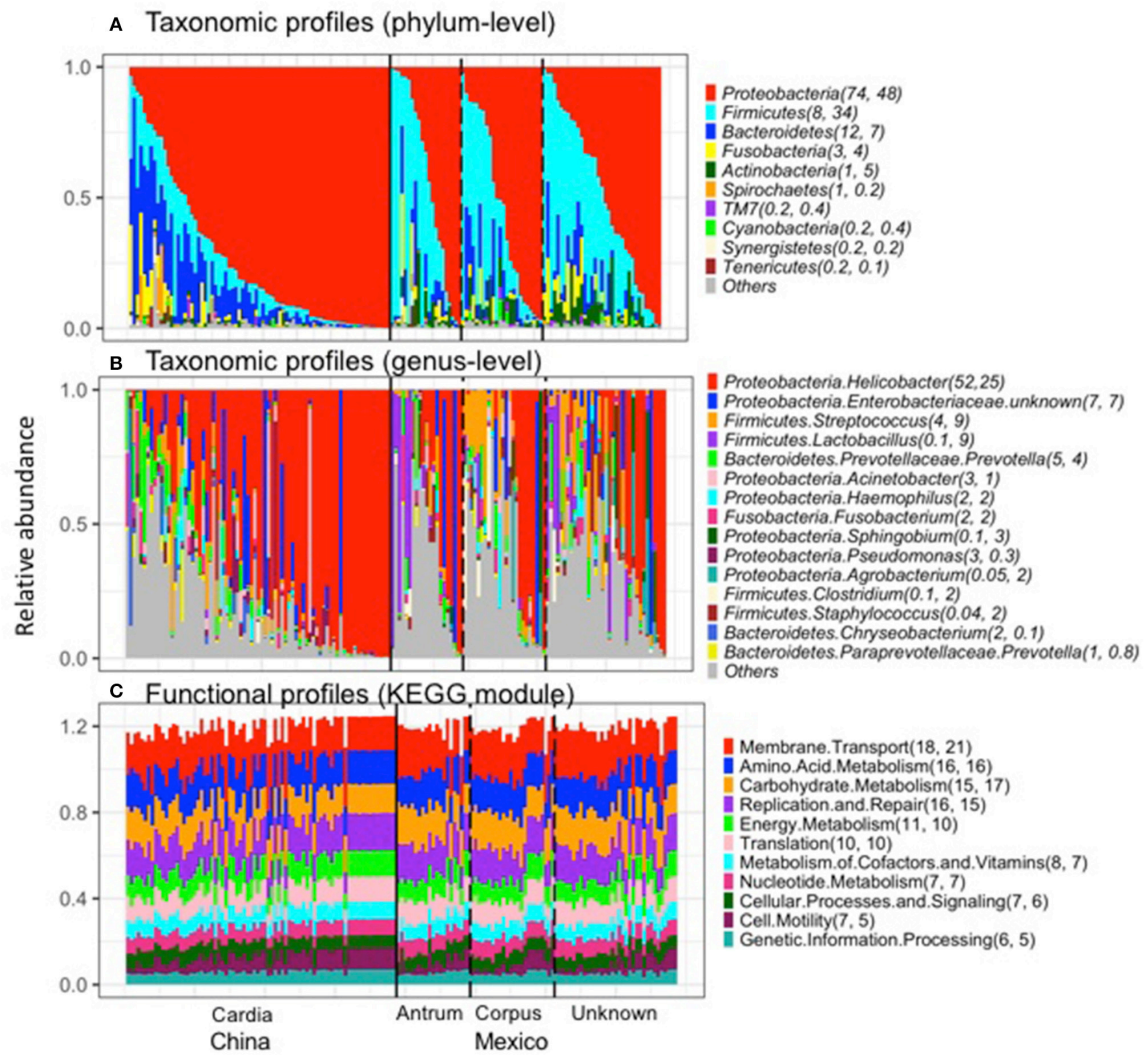
Taxonomic profiles (**A**, Phylum-level; **B**, genus-level) and functional profiles **(C)** of gastric non-malignant tissue microbiota. Each vertical bar represents a unique sample. The numbers in the parenthesis after each phylum/genus/module are the mean relative abundance (%) for Chinese and Mexican samples, respectively. On average, 99% of *Helicobacter* sequence reads were classified as *Hp*. Only the most abundant phyla/genera/modules in Chinese or Mexican samples are shown. All the samples from **(A–C)** are in the same order. The anatomical location and source of the samples are shown at the bottom of the figure.

The taxonomic and functional profiles are shown in Figure 1 for non-malignant tissue samples. According to the non-malignant gastric tissues, the gastric microbiota for both sample sets was mainly composed of *Proteobacteria*, followed by *Bacteroidetes* in Chinese samples or *Firmicutes* in Mexican samples (Figure 1A). The majority of samples from China (78%) and Mexico (50%) were dominated by *Proteobacteria* (relative abundance >50%). Nineteen Mexican samples were dominated by *Firmicutes* and one Chinese sample was dominated by *Bacteroidetes*. The remaining samples did not have a dominant phylum.

The most abundant genus in the non-malignant microbiota of both Chinese and Mexican gastric cancer patients was *Helicobacter* (Figure 1B), and 99% of the *Helicobacter* reads [median (interquartile range): 98.8% (98.7–99.3%)] were classified as *Hp*. As shown in Table 1, the majority of GC patients' stomachs (94% Chinese and 55% Mexican) were colonized by *Hp*, and 53% Chinese and 28% Mexican gastric microbiota were dominated by *Hp* (*Hp* relative abundance >50%).

**Table 1:** *Hp* in GC patients for both nonmalignant and tumor gastric tissue microbiota.

	**China**		**Mexico**	
	**Non-malignant (*n* = 77)**	**Tumor (*n* = 80)**	**Non-malignant (*n* = 80)**	**Tumor (*n* = 54)**
Samples with *Hp* colonization, n (%)	72 (94%)	75 (94%)	44 (55%)	30 (56%)
Samples with relative *Hp* >50%, n (%)	41 (53%)	16 (20%)	22 (28%)	9 (17%)
***HP*** **RELATIVE ABUNDANCE (%)**				
Average	51%	20%	24%	18%
Median (interquartile range)	56% (14–89%)	4% (1–36%)	4% (0–58%)	0 (0–25%)
Maximum	99%	87%	97%	98%

The virtual reconstructed functional profiles (KEGG modules) of non-malignant gastric tissue samples predicted by PICRUSt are shown in Figure 1C. The most abundant module functions in gastric microbiota were membrane transport, amino acid metabolism, carbohydrate metabolism, replication and repair, and energy metabolism in both Chinese and Mexican samples. Compared to the variation in taxonomic profiles, the variation in functional profiles among non-malignant gastric tissue samples was more limited (Figure 1A vs. Figure 1C).

Neither Chinese nor Mexican samples showed an association between gastric microbial features and age or gender (data not shown). Within Mexican samples, no significant difference in microbial alpha diversity, beta diversity and taxa relative abundance for the antrum and corpus non-malignant samples was observed (Supplementary Table 4).

### Comparison of non-malignant gastric tissue to matched tumor tissue

**Figure 2:**
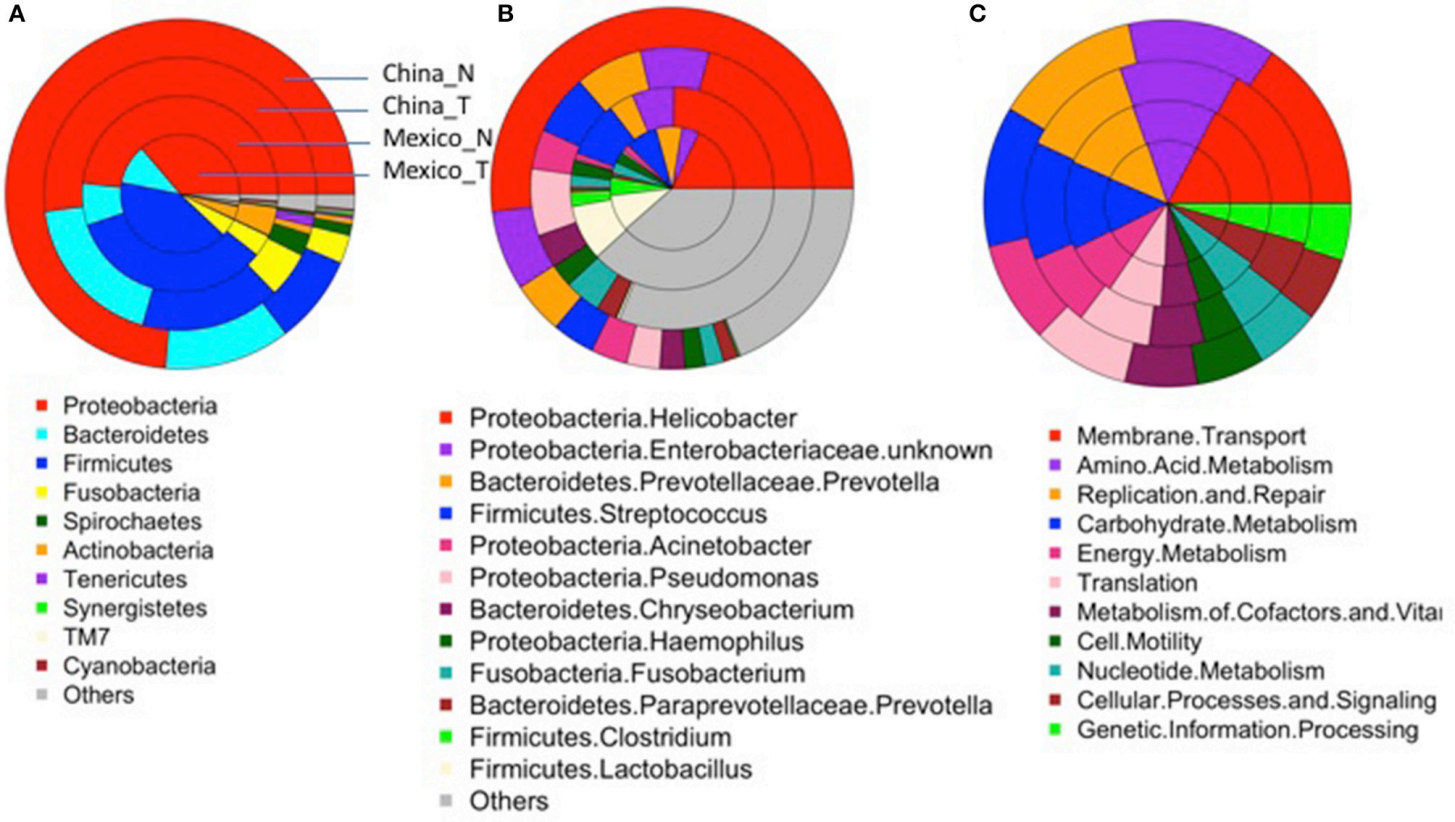
Average taxonomic (**A**, Phylum-level; **B**, genus-level) and functional profiles **(C)** of gastric microbiota in the non-malignant (China_N, Mexico_N) or tumor (China_T, Mexico_T) tissue group. As shown in **(A)**, each donut includes a single sample group. The colors represent different taxonomical/functional groups as shown at the bottom of each figure. The proportion of the color in each donut represents the average relative abundance in each sample group.

After excluding samples with <1,000 reads, 80 tumor tissue samples from China and 54 from Mexico remained for comparison with their matched non-malignant tissues. The taxonomic and functional profiles for these samples are shown in Supplementary Figure 3. The average profiles for both non-malignant and tumor sample groups are shown in Figure 2. Similar to the profiles in non-malignant tissues, the tumor gastric microbiota for both sample sets was also mainly composed of *Proteobacteria*, followed by *Bacteroidetes* in Chinese tumor samples or *Firmicutes* in Mexican tumor samples (Supplementary Figure 3A vs. Figure 2A). The genus with the most abundance in both tumor sample sets was *Helicobacter* (Supplementary Figure 3B vs. Figure 2B) with average relative abundance of 21% in Chinese samples and 18% in Mexican samples. Compared to non-malignant tissues, tumor tissue had less *Proteobacteria*, and higher *Bacteriodetes, Firmicutes, Fusobacteria*, and *Spirochaetes* in Chinese samples. There was no significant change in Mexican samples in phylum-level taxa (Supplementary Table 5). At the genus level, tumor tissue had lower *Helicobacter* abundance relative to non-malignant tissue in both Chinese and Mexican samples. Chinese samples showed substantial differences in alpha diversity as well as several other genus taxa. Mexican samples showed differences in *Clostridia* relative abundance, but did not display differences in alpha diversity measures (Supplementary Table 5). *Hp* relative abundance was also lower in tumor tissues compared to matched non-malignant tissues in both sample sets (Supplementary Table 5). However, the majority of tumor tissues (94% Chinese and 56% Mexican) were colonized by *Hp*, and many tumor samples (20% Chinese and 17% Mexican) were dominated by *Hp* (*Hp* relative abundance >50%; Table 1).

The most abundant module functions in tumor tissues were membrane transport, amino acid metabolism, carbohydrate metabolism, replication and repair, translation, and energy metabolism in both Chinese and Mexican samples (Supplementary Figure 3C, Figure 2C). In both Chinese and Mexican samples, the functional module of infectious disease was higher in non-malignant than in tumor tissues (Supplementary Table 6). Chinese samples showed substantial differences in other functional modules after Bonferroni correction for multiple comparisons (Supplementary Table 6). Mexican samples did not display differences in relative abundance for other functional modules between tumors and non-malignant tissues (Supplementary Table 6). Functional and taxonomic profiles were correlated (Supplementary Figure 4). For example, high infectious disease function was mainly contributed by *Helicobacter* as these factors were positively correlated in relative abundance.

**Figure 3:**
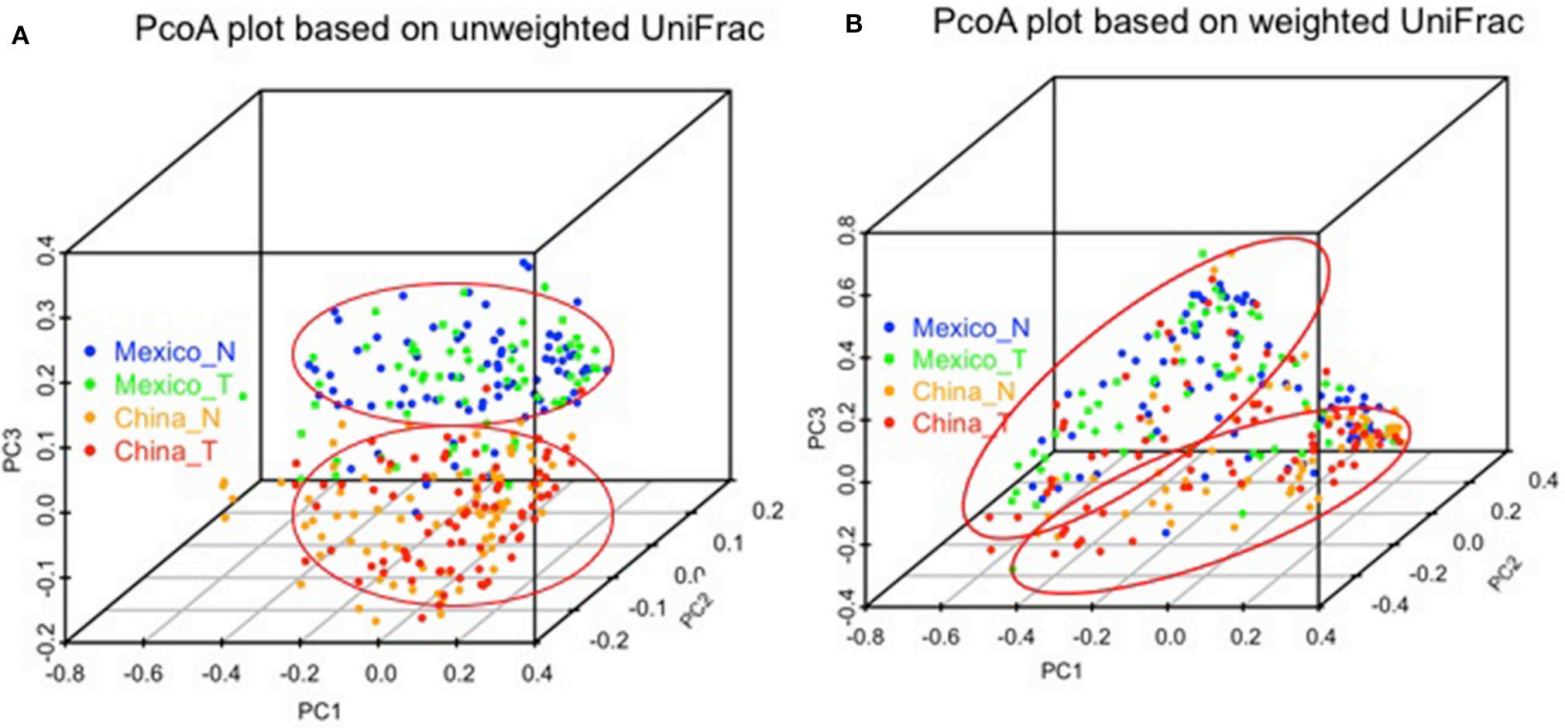
Comparison of gastric microbiota among different sample groups by PCoA plots based on unweighted **(A)** and weighted UniFrac distance **(B)**. The color represents different sample groups as shown in the legend (non-malignant, China_N, Mexico_N; and tumor, China_T, Mexico_T).

We made PCoA plots based on both unweighted or weighted UniFrac distance matrix to visualize similarities and differences among gastric samples. Both plots suggested that gastric samples were primarily clustered by geographic location, rather than by tissue types (Figure 3).

### Comparison of non-malignant gastric tissue to the other body sites

The average relative abundance of the top abundant genera by body sites are shown in Table 2. The top abundant genera are the genera with average relative abundance >0.05 in at least one body site. The top abundant genera in stomach includes *Helicobacter* and an unknown *Enterobacteriaceae* genus in either Chinese or Mexico samples, and two additional genera *Streptococcus and Lactobacillus* in Mexico samples. The top abundant genera in other body sites included *Streptococcus, Prevotella, Haemophilus, Veillonella*, and *Neisseria* in oral cavity, *Lactobacillus* in vagina, *Bacteroides, Faecalibacterium*, and *Alistipes* in stool, *Staphylococcus, Corynebacterium*, and *Propionibacterium* in both skin and nasal cavity. These top genera in each body site were considered as the genera associated with their corresponding body site (e.g., vagina_associated genus refers to *Lactobacillus*). We found that the stomach microbiota was enriched with the genera associated with the oral cavity (combined relative abundance of 17.6 and 11.6% in Mexico and China samples, respectively).

**Table 2:** The average relative abundance of top abundant genera by body sites and their comparison.

	**Stomach_mexico**	**Stomach_china**	**Oral**	**Vagina**	**Stool**	**Skin**	**Nasal**
*Helicobacter*	**0.247**	**0.521**	0.000	0.000	0.000	0.003	0.002
*Enterobacteriaceae.unknown*	**0.067**	**0.072**	0.000	0.000	0.000	0.000	0.000
*Lactobacillus*	**0.089**	0.001	0.000	**0.850**	0.000	0.002	0.002
*Streptococcus*	**0.089**	0.038	**0.294**	0.003	0.000	0.015	0.017
*Prevotella*	0.040	0.047	**0.097**	0.027	0.032	0.005	0.004
*Haemophilus*	0.022	0.018	**0.100**	0.000	0.001	0.002	0.006
*Veillonella*	0.015	0.005	**0.069**	0.000	0.000	0.002	0.002
*Neisseria*	0.010	0.008	**0.054**	0.000	0.000	0.002	0.002
*Bacteroides*	0.003	0.003	0.001	0.001	**0.482**	0.007	0.005
*Faecalibacterium*	0.000	0.000	0.000	0.000	**0.051**	0.002	0.001
*Alistipes*	0.000	0.000	0.000	0.000	**0.054**	0.001	0.000
*Staphylococcus*	0.023	0.000	0.000	0.000	0.000	**0.150**	**0.192**
*Corynebacterium*	0.009	0.001	0.020	0.001	0.000	**0.051**	**0.338**
*Propionibacterium*	0.000	0.000	0.002	0.000	0.000	**0.602**	**0.235**
**COMBINED RELATIVE ABUNDANCE OF THE TOP GENERA IN OTHER BODY SITES**							
Oral_associated	**0.176**	**0.116**					
Nasal_associated	0.032	0.001					
Skin_associated	0.032	0.001					
Stool_associated	0.003	0.003					
Vagina_associated	0.089	0.001					

*The stomach microbiota in this table were based on non-malignant tissue samples only. Top abundant genera refer to genera with relative abundance >0.05 in at least one body site (bolded and highlighted). These top genera in each body site were considered as genera associated with their corresponding body site. For example, oral_associated genera include Streptococcus, Prevotella, Haemophilus, Veillonella, and Neisseria, and their combined relative abundance in stomach samples is the relative abundance of oral_associated genera in the stomach*.

**Figure 4:**
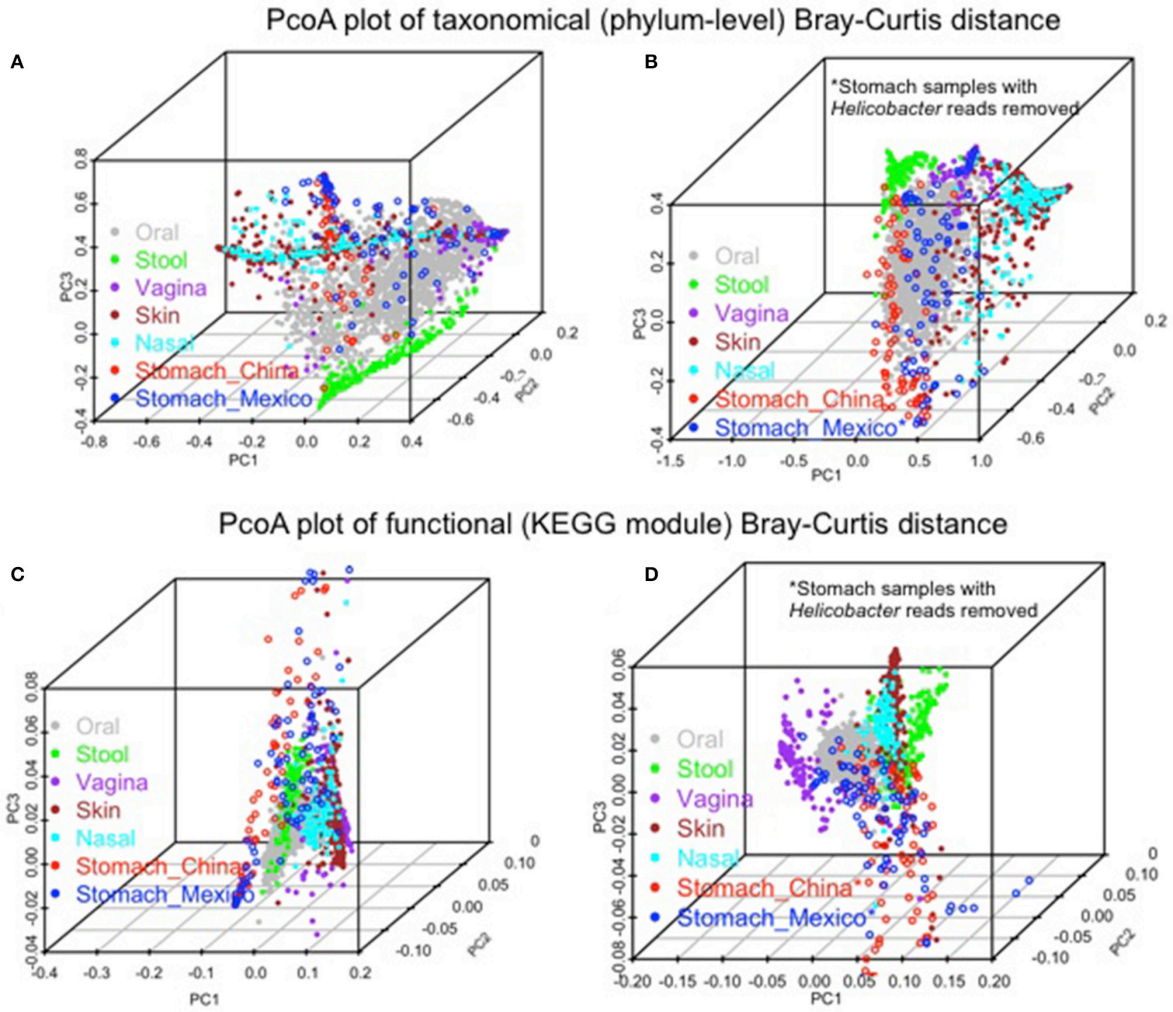
Comparison of the gastric microbiota of the non-malignant tissues with microbiota in the other human body sites (HMP 16S V3–V5 phase 1 data). **(A,B)** PCoA plots of Bray-Curtis distance of phylum-level taxonomic profiles with *Helicobacter* **(A)** or without *Helicobacter* reads **(B)** in stomach samples. **(C,D)** PCoA plots of Bray-Curtis distance of KEGG module profiles with *Helicobacter* **(C)** or without *Helicobacter* reads **(D)** in stomach samples. The color represents different body sites or sample groups as shown in the legend.

Similarities and differences of taxonomic/functional profiles by body sites are shown in Figure 4. The principal coordinates plots based on taxonomic profiles (phylum-level) demonstrated the primary clustering of samples by body sites (Figures 4A,B). The stomach samples, Chinse or Mexico, largely overlapping with oral sample cluster, which was clearer when *Helicobacter* reads were removed (Figures 4A,B). Compared to the principal coordinates plots based on taxonomic profiles, the plots based on functional profiles (KEGG modules) showed a much clearer pattern of clustering by body sites (Figure 4A vs. Figure 4C, Figure 4B vs. Figure 4D). The stomach samples, with or without *Helicobacter* reads removed, either Mexico or Chinese samples, did not cluster with the other body sites, but they also did not cluster with each other as closely as the samples in the other body sites. It suggested higher inter-subject dissimilarity in stomach samples than in the other body sites in functional profiles. The inter-subject dissimilarity by body sites based on Bray-Curtis distance of phylum/KEGG module profiles were then evaluated (Supplementary Figure 5). Mexican stomach samples had the highest inter-subject dissimilarity in phylum profiles. The Chinese samples, however, had inter-subject dissimilarity higher than the other body sites only when *Helicobacter* reads were removed (Supplementary Figure 5A). This might be due to the fact that almost all Chinese samples (94%) had *Helicobacter*. The inter-subject dissimilarity in functional profiles was much higher in the stomachs of both sample sets than in other body sites (Supplementary Figure 5B).

### Comparison of gastric tissue microbiota features by *Hp* colonization status

To further evaluate the gastric tissue microbiota by *Hp* colonization status, we removed the *Helicobacter* reads from the *Hp*+ samples (with *Hp*) and then compared them to the *Hp*− samples (without *Hp*) for alpha diversity, beta diversity, and taxa relative abundance separately for non-malignant and tumor samples. No significant differences were observed among Mexican samples (Supplementary Table 7). A similar comparison could not be performed in the Chinese samples because too few samples were *Hp*− (*n* = 5).

## Discussion

In the largest study of gastric tissue to date, we investigated the gastric microbiota in sets of patients from Mexico and China. In both sets, we showed that *Hp* is the most abundant member of the stomach microbiota, followed by the genera that are commonly seen in the oral microbiota. The principal coordinates plots of Bray–Curtis distance matrix based on phylum-level taxonomic profiles suggested that stomach samples largely overlapped with oral samples. The principal coordinates plots based on functional profiles, however, suggested that stomach microbiota was distinct from the microbiota of other body sites, and had higher inter-subject dissimilarity. We found no differences in microbiota composition by anatomic site or *Hp* status, although we had only limited sample size to detect differences. We did find that the relative abundance of *Hp* was higher in non-malignant than in tumor tissues for both Chinese and Mexican samples.

Gastric microbiota was dominated by phyla *Proteobacteria* in Chinese samples, and by *Proteobacteria* and *Firmicutes* in Mexican samples. This result is consistent with most previous studies based on gastric biopsy, fluid, or tissue in either healthy or cancer cases (Supplementary Table 1). Overall, in cases with high *Hp* relative abundance, the most abundant gastric phylum is *Proteobacteria*, otherwise it is *Firmicutes*.

GC patients, both non-cardia and cardia in Asian populations have been proposed to have chronic gastritis that leads to hypochlorhydria in the stomach (Cavaleiro-Pinto et al., 2011; Sheh and Fox, 2013). Therefore, GC patients have been hypothesized to have diminished or no colonization of *Hp* in the stomach (Sheh and Fox, 2013). Previous studies of gastric microbiota in GC patients were limited, small, and found inconsistent results (summarized in Supplementary Table 1). Studies from Sweden (Eun et al., 2014) and Mexico (Aviles-Jimenez et al., 2014) did not identify *Hp* as the dominant species in any of the samples evaluated, while four other studies in Korea, Taiwan and USA showed results consistent with our finding with *Hp* as the dominant species in GC samples (Dicksved et al., 2009; Eun et al., 2014; Zhang et al., 2015; Tseng et al., 2016). This difference between studies might be due to *Hp* prevalence heterogeneity across study samples. A recent study of 212 chronic gastritis and 103 GC patients in China that used quantitative PCR showed that the bacteria load in the gastric mucosa was increased in cancer patients compared to gastritis patients, and the bacterial load was positively correlated with *Hp* quantity (*R* = 0.38, *P* < 0.001), suggesting *Hp* colonization in GC patients (Wang et al., 2016). In the current study, we found that many Chinese and Mexican GC patients had stomachs dominated by *Hp*. This finding may be relevant to the decision-making of GC treatment. Endoscopic resection has been considered the first line of treatment for early GC in Korea and Japan because it is minimally invasive and effective (Chung et al., 2009; Isomoto et al., 2009). Several studies have shown a benefit for *Hp* eradication in reducing metachronous tumors after resection for early gastric cancer (Fukase et al., 2008; Bang et al., 2015). Our data suggest that the majority of patients diagnosed with GC in these populations have current *Hp* colonization and this may explain why eradication therapy at time of diagnosis may be beneficial.

Our study of gastric microbiota showed less between-sample variation in the functional profiles than in the taxonomic profiles, which is similar to a previous study in other body sites (Human Microbiome Project Consortium, 2012). This finding of less variation in function than in taxa is consistent with functional redundancy across taxa and suggests that taxonomically distinct microbes may have similar functions. Therefore, analysis of functional modules appears to provide insights that analysis of phyla alone may not be able to identify. For example, we showed that stomach microbiota was distinct from microbiotas in other body sites in functional profiles, but not in taxonomical profiles. In addition, inter-subject dissimilarity in functional profiles is much higher in stomach than in other body sites. However, it is important to note that the functional profiles were based on prediction only. Therefore, it is possible that prediction-based biases toward well-documented microbial genomes resulted from exclusion of unknown or poorly documented taxa. Further studies are needed to validate these findings.

Consistent with a study in a United States population (Bik et al., 2006), we did not observe differences in the microbiota between the antrum and corpus in the Mexican GC samples. In contrast, a Chinese study reported that gastritis patients without *Hp* infection had decreased *Prevotella* in the antrum compared to the corpus (Li et al., 2009). Larger studies in subjects without GC are needed to further compare the gastric microbiota by different anatomical sites and also control for population and health conditions.

*Hp* colonization may impact gastric microbiota by induction of host antimicrobial peptides (Hornsby et al., 2008), by directly killing other bacteria through the activity of its own cecropin-like peptide (Putsep et al., 1999), or by inducing physiological changes in host stomach such as pH alteration (Smolka and Backert, 2012), epithelial surface (Wroblewski et al., 2016), gastric hormones and immunologic state (Blaser and Atherton, 2004). However, the difference in the gastric microbiota by host *Hp* colonization status is not fully understood. Consistent with our findings, a study in the United States showed that the relative abundance of non-*Hp* bacteria in *Hp*+ subjects was not altered compared to *Hp*− subjects when *Hp* sequences were eliminated from the analysis (Bik et al., 2006). A study in China however suggested that the major influence of *Hp* on microbiota is the increased bacterial load in the stomach, not the relative abundance of non-*Hp* bacteria groups (Wang et al., 2015). In contrast, a small study of 10 Amerindians and 2 non-Amerindians using the PhyloChip reported marked differences in relative abundance of non-*Hp* bacteria by *Hp* status (Maldonado-Contreras et al., 2011). Our study examined cancer patients, and thus *Hp*− GC patients may have a prior history of *Hp* colonization. Larger studies in subjects without GC using advanced sequencing technology are needed.

As has been previously shown for tumor and matched non-malignant samples from colorectal cancer patients (Burns et al., 2015), we also found taxonomical and functional composition differences between non-malignant and tumor tissues in both Chinese and Mexican cases. This observation might suggest the change of local environment in tumor (e.g., reduction of acid secretion) compared to non-malignant tissues, which leads to *Hp* diminution and corresponding microbial functional changes in tumor. Whether these changes contribute to gastric carcinogenesis or tumor progression require further investigation. Our recent study of the Chinese sample set suggested that the changes in the gastric microbiota including *Hp* relative abundance in non-malignant tissue were associated with cancer risk factors and clinical outcomes including family history of upper gastrointestinal cancer and tumor grades (Yu et al., 2017). Similar associations were, however, not found in the tumor tissues or in the Mexican sample set (data not shown).

While this study includes noteworthy strengths, it also includes limitations. Although, it is the largest study of the gastric non-malignant tissue microbiota from GC patients to date, it includes samples from two different populations with different rates and types of GC. The non-malignant tissue samples were obtained distant but unmeasured from the tumor lesion under sterile conditions and were frozen immediately. Also, unlike most studies of gastric microbiota, we analyzed not only the taxonomic profiles and *Hp* relative abundance, but also virtual reconstructed functional profiles. We compared our gastric tissue data to the HMP data, which included different populations, DNA extraction techniques, and sequencing platforms. We tried to minimize the effects of these differences by restricting our comparisons to the highest and least variable taxonomic level (phylum-level) and functional entity (KEGG module). In addition, a meta-analysis of microbiota studies suggested that differences in microbial populations across body sites are larger than those driven by the experimental protocols, age, geography, and other population characteristics (Lozupone et al., 2013). Another limitation was the use of a DNA extraction method without a bead-beating step. However, we showed that although we may have missed certain bacteria with potentially hard-to-break cell membranes, these bacteria were rare and should not have adversely affected our conclusions. Finally, our study was restricted to cancer patients and we did not have gastric samples from subjects without GC for comparison. Therefore, we cannot evaluate whether results found in GC patients generalize to subjects without GC.

## Conclusions

By analyzing the gastric tissues of two different populations with different types of GC separately, we showed that *Hp* was the dominant taxa in the stomach of many subjects with GC, followed by oral-associated bacteria. Comparison with other body sites suggested that stomach microbiota resembled oral microbiota in phylum-level taxonomical profiles, but not in functional profiles. Our study provided insights of gastric microbiota composition and function in GC patients.

## Author contributions

GY designed the study, analyzed the data and wrote the initial manuscript. JT, NH, RM, RH, MH, LW, CW, TD, PT, and AG performed sample collection and laboratory experiments. GY, JT, JR, PT, CA, and AG contributed to the data interpretation and manuscript revision.

### Conflict of interest statement

The authors declare that the research was conducted in the absence of any commercial or financial relationships that could be construed as a potential conflict of interest.

## Abbreviations

GC: Gastric Cancer
*Hp*: *Helicobacter pylori*
HMP: Human Microbiome Project
NCI: National Cancer Institute
OTUs: Operational Taxonomy Units
QIIME: Quantitative Insights into Microbial Ecology
PD_whole_tree: Phylogenetic diversity
PICRUSt: Phylogenetic Investigation of Communities by Reconstruction of Unobserved States
PERMANOVA: Permutational Multivariate Analysis of Variance.
