# Supplementary material for: Molecular Characterization of the Human Stomach Microbiota in Gastric Cancer Patients
Source: Front Cell Infect Microbiol. 2017 Jul 6;7:302. doi: 10.3389/fcimb.2017.00302 (PMC5498480; doi:10.3389/fcimb.2017.00302)
Supplement: Supplementary file 1 [file Presentation1.pdf]

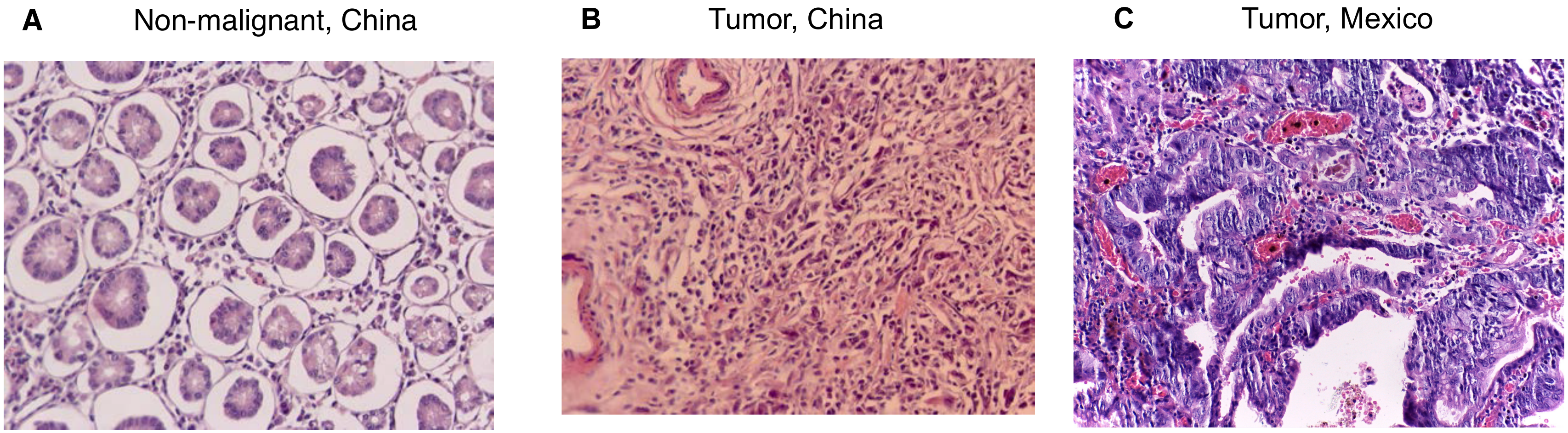

Supplementary Figure 1, Examples of H&E slides from this study.

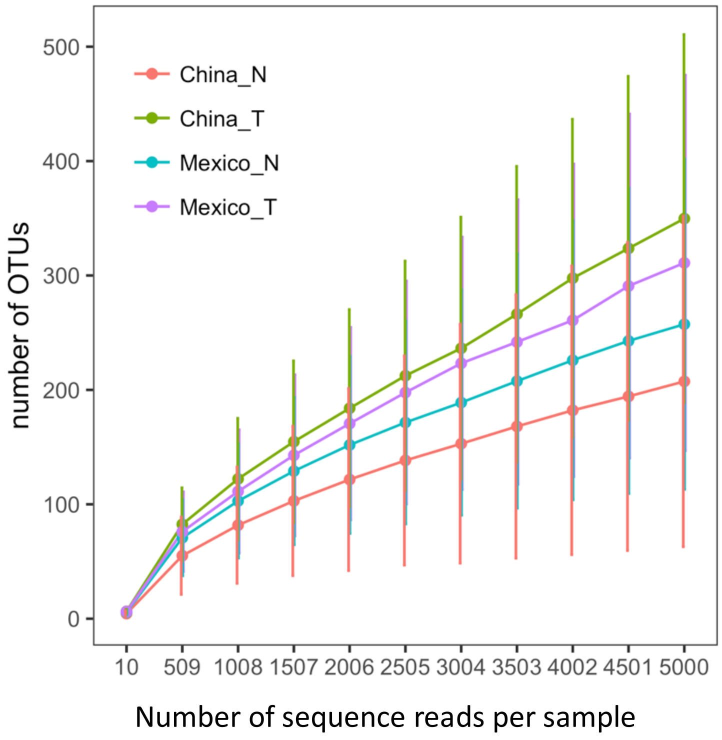

Supplementary Figure 2, Rarefaction curve. Rarefaction curve based on operational taxonomic units (OTUs, 97 similarity). The color represents different sample groups (non-malignant tissue samples in China (China_N) and Mexico (Mexico_N); tumor tissue samples in China (China_T) and Mexico (Mexico_T)). The curves represent mean number of OTUs among each sample group. The bars represent standard deviations.

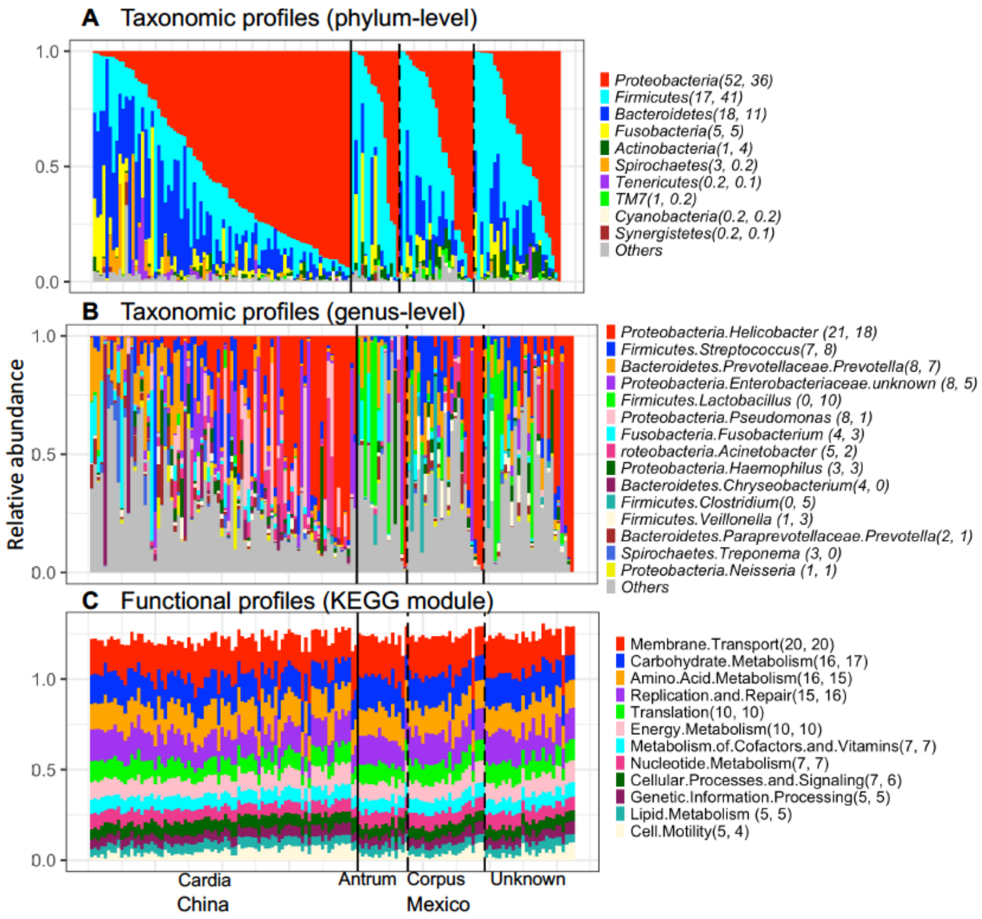

Supplementary Figure 3, Taxonomic profiles (A, Phylum-level; B, genus-level) and functional profiles (C) of gastric tumor tissue microbiota. Each vertical bar represents a unique sample. The numbers in the parenthesis after each phylum/genus/module are the mean relative abundance (%) for Chinese and Mexican samples respectively. All the samples from Figure A, B and C are in the same order. Their anatomical location and sources of the samples are shown at the bottom of panel C.

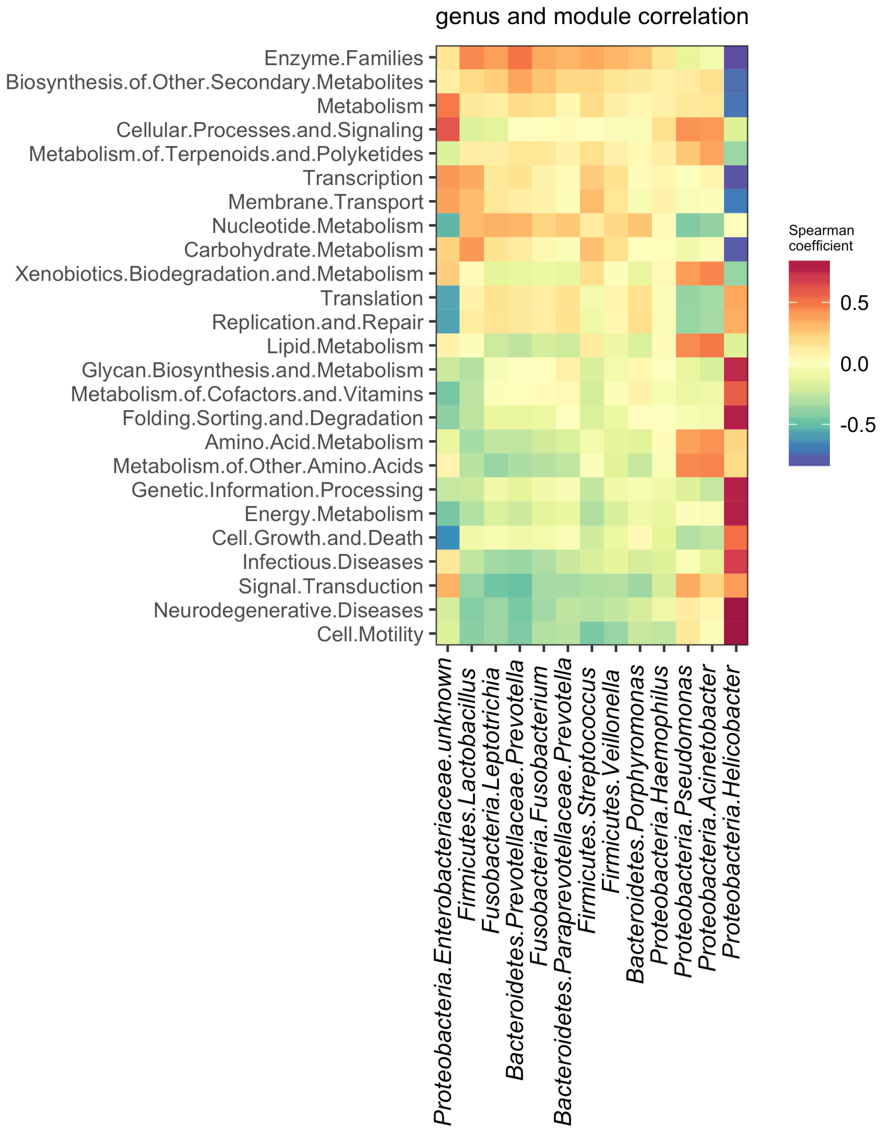

Supplementary Figure 4, Heatmap of Spearman correlation between genus-level taxonomic and genus-level functional profiles among gastric samples. Only the top genera or modules are shown.

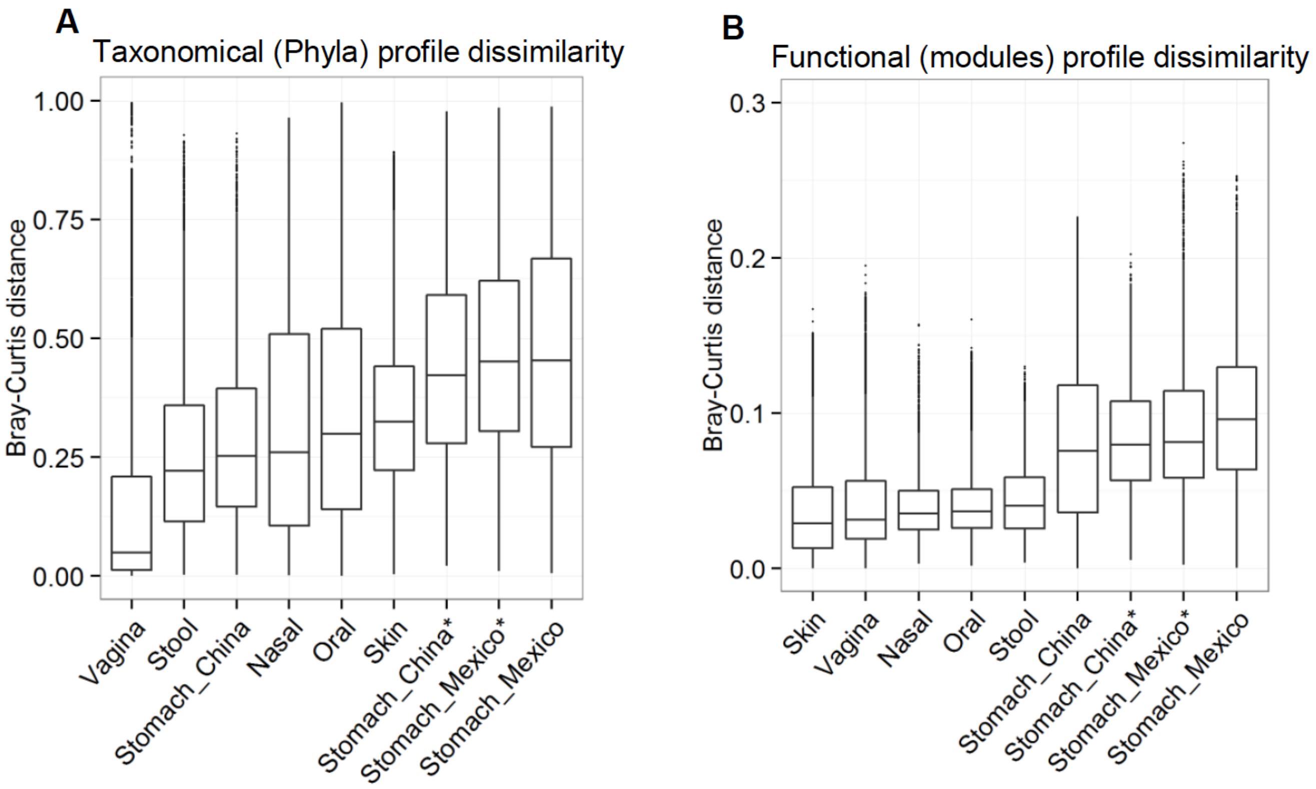

Supplementary Figure 5, Inter-subject dissimilarity by body sites based on Bray-Curtis distance of phylum profiles (A) and KEGG module profiles (B). * stomach samples with *Helicobacter* reads removed. In both figures, boxes are interquartile range (IQR); median values are bands within the boxes; lines outside the boxes are 1.5-times IQR; dots are outliers.

Supplementary Table 1, Summary of recent studies in gastric microbiota

Study subjects			Sample type	Method	Results			
**n**	**Status**	**Race/country**			**top phyla**	***Hp* relative abundance**	**Other dominant genus**	**citation**
Studies without GC cases								
23	healthy	Caucasian, Hispanics and African American/USA	biopsy	Culture + sequencing 16S	*Proteobacteria, Firmicutes, Actinobacteria, Bacteroidetes, Fusobacteria*	*Hp* dominates the *Hp*+ subjects (72% of all clones, up to 99% in some case)	*Streptococcus, Prevotella*	1
6	healthy	Sweden	biopsy	sequencing 16S	*Proteobacteria, Firmicutes, Actinobacteria, Bacteroidetes, Fusobacteria*	*Hp* dominates *Hp*+ subjects (93-97%)	*Streptococcus, Actinomyces, Prevotella, Gemella*	2
45	Gastritis, healthy	Chinese	biopsy	Culture + sequencing 16S	*Firmicutes, Proteobacteria, Actinobacteria, Bacteroidetes, Fusobacteria*	all case were *Hp*-	*steptococcus and prevotella*	3
25	healthy	USA	fluid	sequencing 16S	*Firmicutes, Bacteroidetes, Actinobacteria, Proteobacteria and Fusobacteria*	low (<0.4%)	*Streptococcus, prevotella, Veillonella, Lactobacillus*	4
12	healthy	Spain	biopsy, juice	sequencing 16S	*Firmicutes, Proteobacteria, Actinobacteria*	low	*Streptococcus, Propionibacterium, Lactobacillus and Enterococcus*	5
20	healthy	Colombia	biopsy	sequencing 16S				6
93	ESCC, ESD, and healthy	Iran	biopsy	sequencing 16S	*Proteobacteria*, *Firmicutes*, *Bacteroidetes*, *Actinobacteria Fusobacteria*			7

4	healthy	korean	biopsy, juice	sequencing 16S	*Proteobacteria, Firmicutes, Actinobacteria, Fusobacteria*, and *Bacteroidetes*	66.5% in biopsy and 3.3% in juice		[8]
Studies with GC cases								
63	GC and controls	Korean	biopsy	sequencing 16S	*Proteobacteria*, *Firmicutes*, *Actinobacteria*, *Bacteroidetes*, *Fusobacteria*	*Hp* dominates GC cases (61% in average)	*Streptococcus, Stenotrophomonas, Ralstoni, Prevotella*	[9 10]
15	GC and controls	Mexican	biopsy	Phylo-Chip	*Firmicutes, Proteobacteria, Bacteroidetes, Actinobacteria, Fusobacteria*	low		[11]
27	GC	USA	biopsy	whole genome sequencing		*Hp* dominates GC cases (48% in average, up to 98% in some cases)		[12]
6	GC	Taiwan	tissue	sequencing 16S	*Proteobacteria, Actinobacteria, Firmicutes and Bacteroidetes*	*Hp* dominates GC cases		[13]
15	GC, healthy	Sweden	biopsy	T-RFLP	*Firmicutes, Actinobacteria, Bacteroidetes, Proteobacteria, Fusobacteria*	low	*Streptococcus, Lactobacillus, Veillonella, Prevotella*	[14]
31	GC, metaplasia, gastritis	Korean	biopsy	sequencing 16S		*Hp* dominates GC cases (47% in average, up to 88%)		[15]
12	GC, gastritis	China	Gastric biopsy	qPCR (n=315), sequencing 16S (n=12)	*Proteobacteria, Firmicutes, Bacteroidetes, Fusobacteria Actinobacteria*			[16]

Note: 16S represents 16S rRNA gene; qPCR, quantitative PCR; GC, gastric cancer; ESCC, Esophageal squamous cell carcinoma , ESD, Esophageal squamous dysplasia

References:

1. Bik EM, Eckburg PB, Gill SR, et al. Molecular analysis of the bacterial microbiota in the human stomach. Proc Natl Acad Sci U S A 2006;103:732-7.
2. Andersson AF, Lindberg M, Jakobsson H, et al. Comparative Analysis of Human Gut Microbiota by Barcoded Pyrosequencing. PLoS One 2008;3.
3. Li XX, Wong GLH, To KF, et al. Bacterial Microbiota Profiling in Gastritis without Helicobacter pylori Infection or Non-Steroidal Anti-Inflammatory Drug Use. PLoS One 2009;4.
4. von Rosenvinge EC, Song Y, White JR, et al. Immune status, antibiotic medication and pH are associated with changes in the stomach fluid microbiota. ISME J 2013;7:1354-1366.
5. Delgado S, Cabrera-Rubio R, Mira A, et al. Microbiological Survey of the Human Gastric Ecosystem Using Culturing and Pyrosequencing Methods. Microb Ecol 2013;65:763-772.
6. Yang I, Woltemate S, Piazuelo MB, et al. Different gastric microbiota compositions in two human populations with high and low gastric cancer risk in Colombia. Scientific Reports 2016;6.
7. Nasrollahzadeh D, Malekzadeh R, Ploner A, et al. Variations of gastric corpus microbiota are associated with early esophageal squamous cell carcinoma and squamous dysplasia. Sci Rep 2015;5:8820.
8. Sung J, Kim N, Kim J, et al. Comparison of Gastric Microbiota Between Gastric Juice and Mucosa by Next Generation Sequencing Method. J Cancer Prev 2016;21:60-5.
9. Jo HJ, Kim J, Kim N, et al. Analysis of Gastric Microbiota by Pyrosequencing: Minor Role of Bacteria Other Than Helicobacter pylori in the Gastric Carcinogenesis. Helicobacter 2016.
10. Kim J, Kim N, Jo HJ, et al. An Appropriate Cutoff Value for Determining the Colonization of Helicobacter pylori by the Pyrosequencing Method: Comparison with Conventional Methods. Helicobacter 2015;20:370-80.
11. Aviles-Jimenez F, Vazquez-Jimenez F, Medrano-Guzman R, et al. Stomach microbiota composition varies between patients with non-atrophic gastritis and patients with intestinal type of gastric cancer. Sci Rep 2014;4.
12. Zhang C, Cleveland K, Schnoll-Sussman F, et al. Identification of low abundance microbiome in clinical samples using whole genome sequencing. Genome Biology 2015;16.
13. Tseng CH, Lin JT, Ho HJ, et al. Gastric microbiota and predicted gene functions are altered after subtotal gastrectomy in patients with gastric cancer. Sci Rep 2016;6:20701.
14. Dicksved J, Lindberg M, Rosenquist M, et al. Molecular characterization of the stomach microbiota in patients with gastric cancer and in controls. J Med Microbiol 2009;58:509-16.
15. Eun CS, Kim BK, Han DS, et al. Differences in Gastric Mucosal Microbiota Profiling in Patients with Chronic Gastritis, Intestinal Metaplasia, and Gastric Cancer Using Pyrosequencing Methods. Helicobacter 2014;19:407-416.
16. Wang LL, Zhou JH, Xin YN, et al. Bacterial overgrowth and diversification of microbiota in gastric cancer. European Journal of Gastroenterology & Hepatology 2016;28:261-266.

Supplementary Table 2, Taxa in genus level that were discovered by extraction method with but not without bead-beating step

	Sample 1	Sample 2
Proteobacteria.Rhodobacteraceae.unknown	0.00004	0.00005
Proteobacteria.Enhydrobacter	0.00008	0.00005
Firmicutes.Enterococcaceae.unknown	0.00008	0.00008
Proteobacteria.Sphingobium	0.00004	0.00016
Proteobacteria.Pseudomonas	0.00012	0.00036
Firmicutes.Lactobacillales.unknown	0.00004	0.00055
Proteobacteria.Methylobacterium	0.00050	0.00016
Proteobacteria.Neisseria	0.00008	0.00099
TM7.c__TM7.3.unknown	0.00069	0.00039
Proteobacteria.Paracoccus	0.00127	0.00016
Proteobacteria.Acinetobacter	0.00130	0.00039
Firmicutes.Alicyclobacillus	0.00222	0.00047
Firmicutes.Lachnospiraceae.unknown	0.00004	0.00829
Firmicutes.Gemellaceae.unknown	0.00081	0.02539
**Sum**	0.007	0.037

Note, the values were their relative abundance in each sample

Supplementary Table 3, Raw and filtered sequence reads number for each sample group in non-malignant (N) or tumor (T) tissues

	Qualified reads	Raw reads
China_N	1254585	2095335
China_T	854491	1669393
Mexico_N	842443	1464574
Mexico_T	696330	1092596

Supplementary Table 4, Comparison between antrum and corpus microbiota among Mexican non-malignant tissue samples

	Antrum (n=21)	Corpus (n=24)	P
Alpha diversity			
No. of OTUs	73.7 (62.0-132.7)	100.9 (69.6-125.1)	0.73
Shannon	3.7 (2.1-4.5)	4.2 (2.2-5.0)	0.58
PD_whole_tree	11.0 (8.1-16.6)	12.3 (8.6-15.6)	0.71
Taxa marginally differed by anatomical sites (P values were Bonferroni corrected)			
Acidobacteria	0 (0-0)	0 (0-0.0002)	0.09
Streptococcus (Firmicutes)	0.013 (0.004-0.023)	0.055 (0.025-0.200)	0.10

Note, Median (interquartile range) values are shown. P values were estimated by Wilcoxon rank-sum tests. We estimated the associations of *Hp* colonization with taxa at all taxonomic level. Only taxa with relative abundance >0.1% in 10% samples were included for analysis. Per beta diversity, we found no difference between antrum and corpus by PERMANOVA test (weighted UniFrac: P=0.86, weighted UniFrac: P= 0.31).

Supplementary Table 5, Comparison of alpha diversity and taxa relative abundance between tumor and matched nonmalignant tissue microbiota

China (n=77 pairs)			
	**Non-malignant**	**Tumor**	**P**
Hp relative abundance	0.56 (0.14-0.89)	0.04 (0.01-0.36)	4.00E-06
Alpha diversity			
No. of OTUs	79.00 (43.90-108.60)	112.50 (82.50-158.00)	1.22E-08
Shannon	2.71 (1.10-4.28)	4.26 (3.54-5.29)	6.04E-08
PD_whole_tree	10.68 (7.27-14.23)	13.73 (9.83-19.41)	3.53E-07
Phyla (P values were Bonferroni-corrected, 9 phyla were tested)			
Bacteroidetes	0.04 (0.01-0.17)	0.11 (0.04-0.29)	2.56E-03
Firmicutes	0.05 (0.01-0.12)	0.14 (0.08-0.26)	8.01E-07
Fusobacteria	0 (0-0.01)	0.01 (0-0.06)	5.49E-03
Proteobacteria	0.85 (0.58-0.97)	0.57 (0.21-0.79)	1.29E-07
Spirochaetes	0 (0-0)	0 (0-0.02)	2.71E-05
Genera (P values were Bonferroni-corrected, 60 genera were tested)			
Helicobacter (Proteobacteria)	0.57 (0.14-0.9)	0.04 (0.01-0.36)	1.87E-06
Treponema (Spirochaetes)	0 (0-0)	0 (0-0.02)	1.63E-04
Selenomonas (Firmicutes)	0 (0-0)	0 (0-0.01)	9.58E-04
Fusobacterium (Fusobacteria)	0 (0-0.01)	0.01 (0-0.03)	4.45E-03
Streptococcus (Firmicutes)	0.01 (0-0.04)	0.03 (0-0.08)	1.26E-02
Gemellaceae.unknown (Firmicutes)	0 (0-0)	0 (0-0.01)	1.62E-02
Pseudomonas (Proteobacteria)	0 (0-0.02)	0.01 (0-0.1)	2.56E-02
Paraprevotellaceae.Prevotella (Bacteroidetes)	0 (0-0.01)	0.01 (0-0.02)	4.27E-02

Mexico (n=54 pairs)			
	**Non-malignant**	**Tumor**	**P**

Hp relative abundance	0.01 (0-0.60)	0 (0-0.25)	0.01
Alpha diversity			
No. of OTUs	100.80 (62.85- 134.00)	103.70 (72.34-141.20)	0.37
Shannon	4.27 (2.46-4.80)	4.47 (2.87-5.08)	0.18
PD_whole_tree	11.61 (8.64-16.77)	11.74 (9.19-15.72)	0.98
Taxa relative abundance (P value was Bonferroni-corrected, 26 class were tested)			
Clostridia (Firmicutes)	0.09(0.01-0.15)	0.1(0.03-0.29)	0.04

Note, Median (interquartile range) values are shown. P values were estimated by Wilcoxon signed-rank tests; Only taxa with relative abundance >0.1% in 10% Chinese or Mexican samples were included for analysis. For Chinese samples, only phyla and genera significantly differed by tissue type are shown. For Mexican samples, only class *Clostridia* is shown because it is the only one out of all taxa tested that significantly differed by tissue type. The phylum name of each genus is shown in the parenthesis. Unknown represents unclassified reads at genus level (e. g. *Gemellaceae.unknown* represents unclassified reads in *Gemellaceae*).

Supplementary Table 6, Functional difference in module relative abundance between tumor and matched nonmalignant tissue microbiota

China (n=77 pairs)			
	**Non-malignant**	**Tumor**	**P**
Cancers	0.004(0.003-0.004)	0.002(0.002-0.003)	2.11E-07
Metabolic.Diseases	0.001(0.001-0.001)	0.001(0.001-0.002)	4.90E-07
Neurodegenerative.Diseases	0.011(0.006-0.013)	0.005(0.004-0.009)	5.69E-07
Circulatory.System	0.002(0.001-0.003)	0.001(0.000-0.002)	2.79E-06
Enzyme.Families	0.030(0.028-0.032)	0.032(0.030-0.035)	2.87E-06
Digestive.System	0.000(0.000-0.001)	0.001(0.001-0.001)	3.50E-06
Energy.Metabolism	0.111(0.096-0.121)	0.096(0.089-0.105)	3.91E-06
Infectious.Diseases	0.010(0.009-0.010)	0.008(0.007-0.009)	6.26E-06
Signaling.Molecules.and.Interaction	0.006(0.004-0.007)	0.004(0.003-0.005)	6.99E-06
Cell.Motility	0.079(0.050-0.096)	0.050(0.034-0.070)	1.41E-05
Poorly.Characterized	0.077(0.069-0.087)	0.087(0.082-0.092)	1.70E-05
Biosynthesis.of.Other.Secondary.Metabolites	0.008(0.007-0.011)	0.011(0.010-0.013)	3.37E-05
Glycan.Biosynthesis.and.Metabolism	0.057(0.046-0.062)	0.047(0.041-0.052)	3.94E-05
Immune.System	0.002(0.001-0.002)	0.001(0.001-0.002)	6.91E-05
Transcription	0.029(0.022-0.038)	0.036(0.033-0.040)	7.45E-05
Endocrine.System	0.003(0.002-0.004)	0.004(0.003-0.005)	7.64E-05
Genetic.Information.Processing	0.063(0.050-0.071)	0.051(0.048-0.056)	8.24E-05
Folding..Sorting.and.Degradation.	0.053(0.044-0.058)	0.046(0.043-0.050)	1.06E-04
Environmental.Adaptation	0.004(0.002-0.004)	0.003(0.002-0.003)	1.46E-04
Metabolism	0.037(0.032-0.042)	0.041(0.038-0.045)	1.69E-04
Metabolism.of.Cofactors.and.Vitamins	0.079(0.074-0.081)	0.074(0.069-0.078)	3.46E-04
Carbohydrate.Metabolism	0.146(0.137-0.157)	0.156(0.147-0.162)	9.13E-04
Transport.and.Catabolism	0.003(0.003-0.004)	0.004(0.004-0.005)	9.05E-03
Membrane.Transport	0.170(0.159-0.199)	0.196(0.179-0.210)	9.24E-03

Nervous.System	0.001(0.001-0.001)	0.001(0.001-0.001)	1.10E-02
Cell.Growth.and.Death	0.011(0.009-0.011)	0.010(0.008-0.011)	2.42E-02
**Mexico (n=54 pairs)**			
	**Non-malignant**	**Tumor**	**P**
Infectious.Diseases	0.008(0.008-0.010)	0.008(0.007-0.009)	3.49E-03

Note, Median (interquartile range) values are shown. P values were estimated by Wilcoxon signed-rank tests and Bonferroni corrected. A total of 41 functional modules were compared between tumor and nonmalignant tissues and only the modules showing significant differences are shown.

Supplementary Table 7, Microbial alpha diversity by *Hp* colonization status

Non-malignant Tissues				
	China	Mexico		
	Hp+ (n=55)	*Hp*- (n=36)	*Hp*+ (n=35)	P
No. of OTUs	136.3(102.5-170.8)	130.2(88.9-152.5)	114.6(86.4-147.6)	0.45
Shannon	5.1(4.3-5.6)	4.6(3.8-5.2)	4.73(3.9-5.1)	0.9
PD_whole_tree	15.7(11.6-20.4)	13.7(9.0-17.5)	13.9(9.3-17.1)	0.98
Tumor tissues				
	China	Mexico		
	Hp+ (n=74)	*Hp*- (n=31)	*Hp*+ (n=22)	P

No. of OTUs	123.1(97.0-158.1)	117.7(91.9-153.8)	147.1(86.7-164.3)	0.65
Shannon	4.9(4.1-5.4)	4.8(4.3-5.3)	5.3(3.4-5.6)	0.54
PD_whole_tree	14.0(10.6-19.2)	12.9(10.7-17.1)	15.2(11.0-18.4)	0.52

Note: *Hp*+ indicates samples with *Hp* colonization, and *Hp*- indicates samples without *Hp* colonization. All the microbial measurements were estimated after removing all *Helicobacter* reads from analysis. Only samples with at least 1000 reads after removing *Helicobacter* reads were included for analysis. P-values were estimated by Wilcoxon rank-sum tests. *Hp*- Chinese samples are not shown due to the limited sample size (n=5). No significant difference in taxa relative abundance and beta diversity by *Hp* colonization status was observed (data not shown).
